# Doped and undoped graphene platforms: the influence of structural properties on the detection of polyphenols

**DOI:** 10.1038/srep20673

**Published:** 2016-02-10

**Authors:** Chu’Er Chng, Zdenek Sofer, Martin Pumera, Alessandra Bonanni

**Affiliations:** 1 Division of Chemistry & Biological Chemistry, School of Physical and Mathematical Sciences, Nanyang Technological University, Singapore 637371; 2 Department of Inorganic Chemistry, Institute of Chemical Technology, 166 28 Prague 6, Czech Republic

## Abstract

There is a huge interest in doped graphene and how doping can tune the material properties for the specific application. It was recently demonstrated that the effect of doping can have different influence on the electrochemical detection of electroactive probes, depending on the analysed probe, on the structural characteristics of the graphene materials and on the type and amount of heteroatom used for the doping. In this work we wanted to investigate the effect of doping on graphene materials used as platform for the detection of catechin, a standard probe which is commonly used for the measurement of polyphenols in food and beverages. To this aim we compared undoped graphene with boron-doped graphene and nitrogen doped graphene platforms for the electrochemical detection of standard catechin oxidation. Finally, the material providing the best electrochemical performance was employed for the analysis of real samples. We found that the undoped graphene, possessing lower amount of oxygen functionalities, higher density of defects and larger electroactive surface area provided the best electroanalytical performance for the determination of catechin in commercial beer samples. Our findings are important for the development of novel graphene platforms for the electrochemical assessment of food quality.

Heteroatom doped graphene has been lately considered as an ultimate candidate for numerous applications due to the possibility to tailor the material characteristics and to improve the physicochemical, optical, structural and electronic properties^1^,^2^,^3^,^4^,^5^,^6^,^7^,^8^. It has been recently demonstrated that heteroatom doping can endow graphene materials with improved electrochemical properties^9^,^10^,^11^. The effect of doping on the electroanalytical performance of graphene platforms has been investigated for various dopant types and concentrations, and it has been shown that both p-type and n-type graphene can provide an improved electrochemical response depending on the different application^12^,^13^,^14^,^15^. In fact, it was found that doping with heteroatoms with different electronegativity can favour the thermodynamic interaction between the graphene platform and the analysed probe, thus providing an enhanced electroanalytical signal^12^,^15^. Parallel to doped graphene, a comparison with undoped material should always be performed when studying the effect of doping on the behaviour of a graphene electrochemical platform. Specifically, the material characteristics such as amount of oxygen functionalities, presence of defects and value of surface area should be carefully evaluated in order to establish whether the increased response is due to the former properties or to the kind and amount of dopant. To date, a very limited number of studies provide a comprehensive investigation on these aspects. Hence, there is urgent need for more systematic studies in which all material and analyte features are taken into account.

In this work we investigate the effect of heteroatom doping on the detection of catechin, a polyphenol generally used as an index of food and beverage quality. A part from traditional techniques based on tedious and expensive chromatographic analysis^16^,^17^,^18^, catechin has been also detected by electrochemistry, using carbon platforms such as single-walled and multi-walled carbon nanotubes^19^,^20^. To the best of our knowledge there are no studies in the literature reporting the electrochemical detection of catechin on doped-graphene materials. In this study, we employ two graphene platforms doped with heteroatoms showing different electronegativity namely boron doped graphene (p-type doping) and nitrogen doped graphene (n-type doping), and we compared their electrochemical performance with that of a thermally reduced undoped graphene for the detection of catechin. We chose for the comparison an undoped material with specific structural characteristics such as low concentration of oxygen functionalities (given by a high C/O ratio from XPS analysis), large amount of structural defects (corresponding to low D/G ratio obtained by Raman spectroscopy) and large electroactive surface area. We wanted to address the question whether the presence of dopant could still provide an enhanced electrochemical performance as compared to the chosen undoped graphene.

We found that, for the examined case, the best electroanalytical response was provided by the undoped graphene which was the material possessing the highest C/O ratio and the largest D/G ratio and electroactive surface area as compared to both heteroatom doped graphene materials. This opens new possibilities in the choice of the best suited graphene platform for electrochemical applications.

## Experimental

### Materials and Apparatus

Glassy carbon (GC) electrodes, (diameter = 3 mm), Ag/AgCl reference electrode and platinum counter electrode were obtained from CH Instruments (Austin, TX, USA). Boron – doped diamond electrode with a doping level of 1000 ppm of B and an H terminated surface was purchased from Windsor Scientific.

Graphite was provided by Asbury Carbons. Fuming nitric acid (>90%) was purchased from by J.T. Baker. Sulfuric acid (95–98%), potassium chlorate (98%), hydrogen peroxide (3%), *N,N-*dimethylformamide (DMF), hydrochloric acid (37%), ethanol, sodium hydroxide, sodium chloride, sodium phosphate monobasic, sodium nitrate, potassium phosphate dibasic, potassium permanganate, potassium chloride, and (+)-catechin hydrate were purchased from Sigma Aldrich (Singapore). Milli-Q water (resistivity: 18.2 MΩcm) was used throughout the experiments. Beer samples were obtained from a local supermarket. Sample n.1 and sample n.2 are lager beers from different brands whilst sample n.3 is a stout beer.

A μAutolab type III electrochemical analyzer (Eco Chemie, The Netherlands) connected to a personal computer and controlled by General Purpose Electrochemical Systems, GPES Version 4.9 software (Eco Chemie) was used to perform differential pulse voltammetry measurements.

A Thermoscientific Finnpipette (volume range 1–10 μl, imprecision on 1 μl below 8%) was employed for the electrode modification with graphene materials.

### Preparation of Thermally Reduced Graphene

Thermally reduced graphene was prepared first by synthesising graphite oxide through the Staudenmaier method^21^ before thermal exfoliation/reduction at 1050 °C was performed. Graphite oxide was obtained by adding 27 mL of nitric acid (98%) and 87.5 mL of sulphuric acid (98%) into a flask containing a magnetic stir bar. The mixture was cooled to 0 °C before the addition of 5 g of graphite. To ensure homogeneous dispersion and to avoid agglomeration, the mixture was stirred vigorously. Subsequently, 55 g of potassium chlorate was added slowly to the mixture at the maintained temperature of 0 °C. Once potassium chlorate was completely dissolved, the cap of the flask was loosened to allow any produced gas to escape. The mixture was stirred continuously for 72 hours at room temperature for a complete reaction. After which, the mixture was decanted and poured into 3 L of distilled water. The formed graphite oxide was then dispersed in hydrochloric acid (5%) and repeated centrifugation and re-dispersion into distilled water was performed with silver nitrate and barium nitrate until there was a negative reaction to chloride and sulphate ions. Finally, the obtained slurry was dried at 50 °C for 48 hours in a vacuum oven.

Thermally reduced graphene was prepared by introducing 0.2 g of graphite oxide into a quartz capsule connected to a magnetic manipulator, which was inside a vacuum tight tube furnace with controlled atmosphere. The magnetic manipulator created a temperature gradient of over 1000 °C min^−1^. The sample was flushed with nitrogen repeatedly before it was inserted by the magnetic manipulator into the preheated furnace and held for 3 minutes. The nitrogen flow rate was maintained at 1000 °C min^−1^ to remove any exfoliation by-products from the procedure.

### Preparation of boron-doped graphene (BDG)

Boron-doped graphene (BDG) was prepared from graphite oxide synthesized through Staudenmaier method. Graphite oxide was thermally exfoliated in the presence of a boron precursor, namely boron trifluoride diethyl etherate (BF_3_Et_2_O). Exfoliation was performed in a bubbler filled with the liquid boron precursor at 20 °C and 1000 mbar. Nitrogen carrier gas with a flow rate of 100 mL/min was used and dilution was performed with 1 L/min nitrogen and hydrogen/nitrogen mixture (0.5 L/min N_2_ and 0.5 L/min H_2_). The reactor was continuously flushed with nitrogen and the flow of boron precursor was stabilised for 5 minutes before it was introduced into the hot region of the reactor. Exfoliation was then performed for 12 minutes at 1000 °C.

### Preparation of nitrogen-doped graphene (NDG)

Nitrogen-doped graphene was prepared from graphite oxide synthesized through Hummers method^22^ before exfoliation was performed under ammonia atmosphere.

Graphite oxide was prepared by adding 2.5 g of sodium nitrate, 5 g of graphite and 115 mL of sulphuric acid (98%) into a flask under continuous stirring. The mixture was cooled in an ice bath before the addition of 15 g of potassium permanganate. Vigorous stirring was maintained for 2 hours to obtain a homogenous solution. After that, the mixture was cooled down and then reheated to 35 °C for 30 minutes. Subsequently, the mixture was diluted with 250 mL of deionised water and it was further heated to 70 °C. The temperature of the mixture was maintained for 15 minutes before it was further diluted with 1000 mL of deionised water. The removal of unreacted manganese dioxide and potassium permanganate was carried out by adding hydrogen peroxide (3%) into the mixture and decanting. Repeated centrifugation and redispersion into distilled water was performed with barium nitrate until a negative reaction to sulphate ions was observed. Graphite oxide slurry was then dried at 60 °C for 48 hours in a vacuum oven.

**Figure 1:**
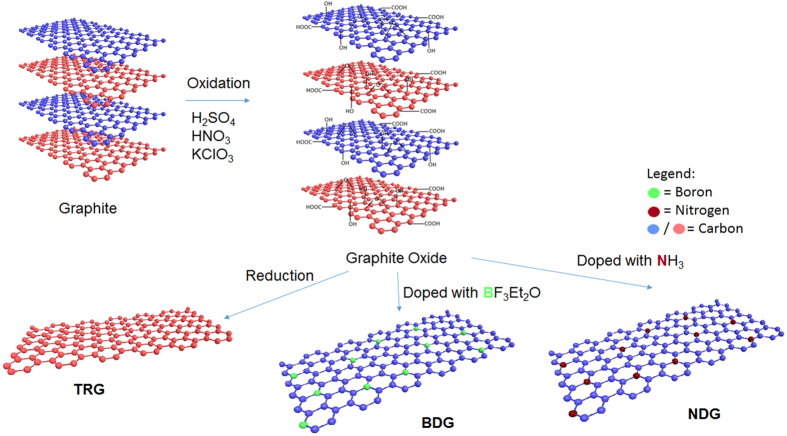
Schematic of the preparation of undoped and doped graphene materials.

Nitrogen-doped graphene was prepared by exfoliation of produced graphite oxide in ammonia atmosphere. A quartz glass capsule was filled with 100 mg of graphite oxide before it was connected to a magnetic manipulator and placed in a horizontal quartz glass reactor. The reactor was flushed continuously with nitrogen before it was introduced into the hot region. Then the nitrogen flow was changed to ammonia. The temperature of the mixture was maintained for 12 minutes at 600 °C and the ammonia flow rate of 300 mL/min was used to remove any exfoliation by-products. Fig. 1. shows a schematic of the preparation of undoped and doped graphene materials.

### Electrochemical measurements

The synthesised thermally reduced graphene (TRG), boron-doped graphene (BDG) and nitrogen-doped graphene (NDG) were ultrasonicated for few minutes before each use. After ultrasonication, 1 μL of the material was deposited onto the surface of a glassy carbon (GC) working electrode. The solvent was left to evaporate at room temperature in order to obtain a randomly distributed film of the desired material on the electrode surface. After each measurement the surface of GC was cleaned by polishing with 0.05 μm alumina powder on a polishing cloth.

Electrochemical experiments were performed in a 4 mL voltammetric cell at room temperature (25 °C) using a three electrode configuration.

Differential pulsed voltammetry parameters used for the experiment were applied as follows: 3 s equilibration time, 50 ms modulation time, 0.5 s interval time, 25 mV modulation amplitude, and 4 mV step. The raw data obtained were treated by a baseline correction with a peak width of 0.01, using GPES software. Measurements were performed in a 4 mL solution containing various concentrations of standard analyte in 100 mM phosphate buffer solution (PBS) at pH 7.3. Catechin hydrate in increasing concentrations from 1.2 μM to 12.0 μM was used for the measurements, similarly to previous findings^23^.

The analysis of commercial lager beer sample was performed by using a dilution factor of 1:10. Standard addition method was used for the analysis of real samples.

## Results and Discussion

In this study we wanted to compare the electrochemical performance of undoped and doped graphene platforms namely thermally reduced graphene (TRG), boron doped graphene (BDG), and nitrogen doped graphene (NDG) for the detection of catechin, an important polyphenol which is correlated to food quality. Unmodified glassy carbon (GC) electrode and boron doped diamond (BDD) electrode were also used as reference materials. We wanted to investigate if the presence of heteroatoms with different electronegativity could have an influence on the electrochemical response provided by the graphene platform, as it could be expected from previous works^12^,^13^,^14^,^15^,^24^,^25^, or if the material characteristics would play a major role.

In order to gain more insight into the material properties, characterization was performed by XPS, Raman spectroscopy and prompt gamma-activation analysis^25^ and the results were collated in Table 1. The C/O ratio provided by XPS analysis gives an indication on the amount of oxygen functionalities which are present on the material surface, being the higher C/O ratio indicative of a lower amount of oxygen containing groups. From the obtained results we can conclude that a larger amount of oxygen functionalities is present on NDG surface, followed by BDG and finally TRG (for the detailed XPS spectra please refer to Figure S1 in Supporting Information). Raman characterization provides information on the structural disorders on the material surface. The D band at around 1350 cm^−1^ is correlated to the presence of defects due to sp^3^ hybridized carbon whilst the G band at around cm^−1^ 1560 indicates the sp^2^ hybridized carbon. The ratio between the intensities of D and G band provide information on the degree of disorders in the carbon structure of the material. As depicted in Table 1, a larger amount of defects is present on TRG surface while BDG is the material containing less structural disorders. In addition, SEM characterization confirmed the successful thermal exfoliation of all graphene materials, showing a typical exfoliated structure (see Figure S3, Supporting Information).

Moreover, from a previous electrochemical study to evaluate the electroactive surface area of all materials, the following results were obtained: 2.28 × 10^−2^ cm^2^ for GC, 2.58 × 10^−2^ cm^2^ for BDD, 4.72 × 10^−2^ cm^2^ for BDG, 8.62 × 10^−2^ cm^2^ for NDG and 1.78 × 10^−1^ cm^2^ for TRG^25^. The values of heterogeneous electron transfer (HET) rates were also evaluated using Nicholson method^26^. The calculated HET constant were: *k*^0^ = 2.30 × 10^−4^ cm s^−1^, *k*^0^ = 2.03 × 10^−4^ cm s^−1^, *k*^0^ = 1.73 × 10^−2^ cm s^−1^, *k*^0^ = 6.49 × 10^−2^ cm s^−1^, *k*^0^ = 4.63 × 10^−2^ cm s^−1^ for GC, BDD, BDG, NDG, and TRG respectively. To summarize the characterization results, TRG is the material with lowest content of oxygen functionalities, highest amount of structural disorders and largest electroactive surface area.

**Figure 2:**
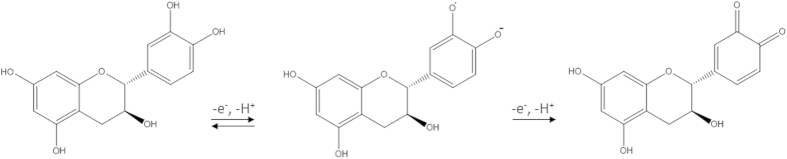
Electrochemical oxidation of catechin.

The oxidation of catechin occurs sequentially at the catechol and resorcinol group respectively^27^. The first oxidation is a reversible process taking place at the catechol 3′, 4′ - dihydroxyl electron-donating groups, while the second oxidation is an irreversible process occurring at the hydroxyl group of resorcinol group (see Fig. 2).

The oxidation process is pH dependant, with a shift towards lower oxidation potentials when the pH of the solution is increased from 3.5 to 8.0^28^. For this reason the measurement was performed in the higher pH range, in order for the oxidation to occur at lower potentials, which in turn contributes to a better selectivity for real sample analysis.

**Figure 3:**
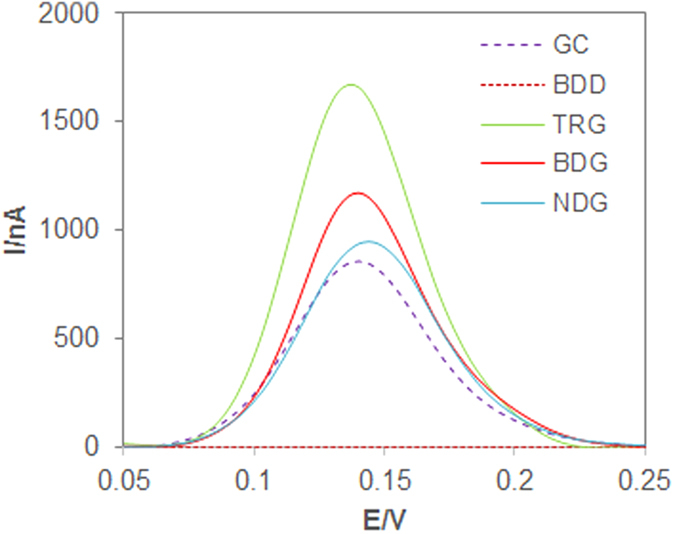
DPV profiles for the determination of catechin at concentrations of 12.0 μM on glassy carbon (GC), boron doped diamond (BDD), thermally reduced graphene (TRG), boron doped graphene (BDG) and nitrogen doped graphene (NDG) electrodes. Conditions: 100 mM phosphate buffer, pH 7.3; step potential 4 mV, modulation amplitude 25 mV.

**Figure 4:**
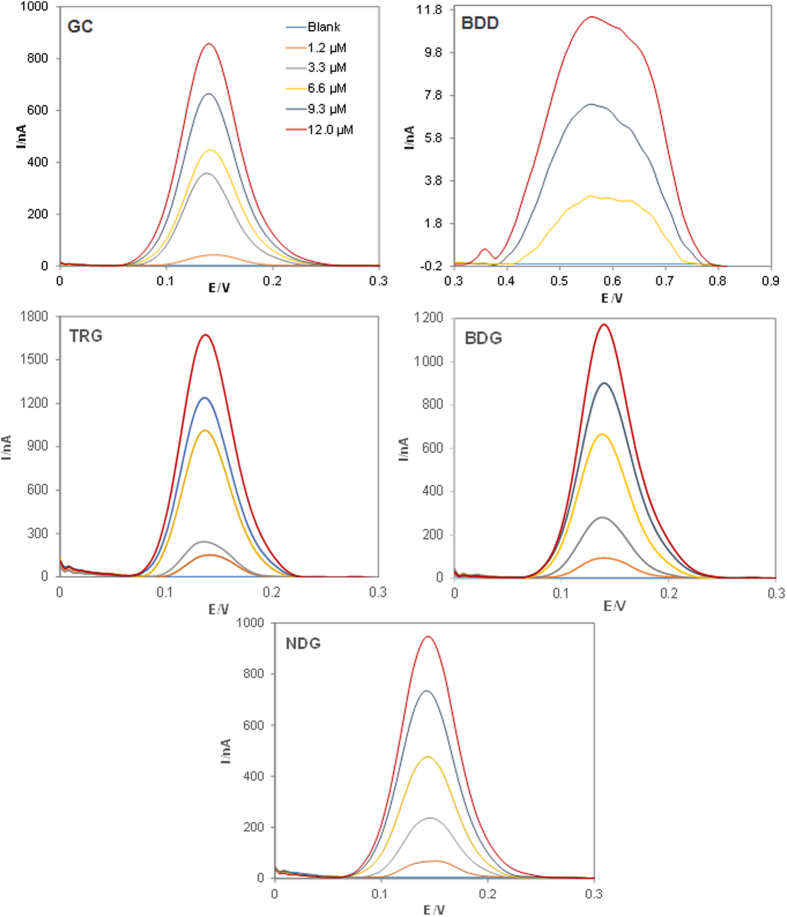
DPV profiles for the determination of catechin at concentrations from 0 μM (blank) to 12.0 μM on glassy carbon (GC), boron doped diamond (BDD), thermally reduced graphene (TRG), boron doped graphene (BDG) and nitrogen doped graphene (NDG) electrodes. Conditions: 100 mM phosphate buffer, pH 7.3; step potential 4 mV, modulation amplitude 25 mV.

Figure 3 shows a preliminary study comparing the oxidation peaks of catechin on the five different materials for 12.0 μM concentration, and Table 2 shows the collated data from Fig. 3. With reference to Fig. 3 and Table 2 all graphene materials, either doped or undoped, show an improved electrochemical response in terms of peak intensity when compared to GC bare electrode, while the oxidation potential is similar for all materials including GC. On the other hand, BDD shows a poorer response in terms of both peak intensity and peak potential. In fact, the oxidation of catechin on BDD happens at a much higher potential as compared to the rest of materials, as also depicted in Fig. 4.

The observed trend could be attributed to the structure of BDD which contains sp^3^ hybridized carbon and therefore lacks of the sp^2^ network that would be necessary to form π-π stacking interactions with the aromatic polyphenol used as probe. Such interactions, which are very likely to occur on both doped and undoped graphene materials, are able to promote an accelerated heterogeneous electron transfer^12^.

**Figure 5:**
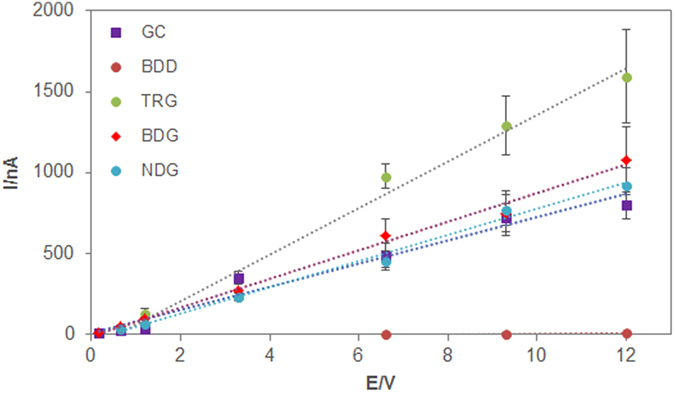
Calibration curve of catechin on different electrode materials: glassy carbon (GC), boron doped diamond (BDD), thermally reduced graphene (TRG), boron doped graphene (BDG) and nitrogen doped graphene (NDG) electrodes.

The response from the oxidation of catechin on bare GC, BDD, TRG, BDG and NDG was studied between 1.2–12.0 μM and the voltammograms were displayed in Fig. 5. Calibration curves of peak current (nA) versus concentration (μM) were plotted to study the sensitivity, selectivity and linearity of the response of each material towards the oxidation of catechin. The slope of calibration curve, the correlation coefficient (R^2^) and the peak width at half height (W_1/2_) are consolidated in Table 3. From the extracted data, the calibration sensitivity for the oxidation of catechin is the highest at 143.22 nA μM^−1^ on TRG, followed by BDG at 88.283 nA μM^−1^, NDG at 81.282 nA μM^−1^, GC at 68.267 nA μM^−1^ and finally BDD at 1.6946 nA μM^−1^.

Overall, both undoped and doped graphene materials showed enhanced sensitivity on the detection of catechin as compared to bare GC, whilst among the doped graphenes, BDG showed a better sensitivity than NDG. All materials presented good linearity of response with R^2^ ≥ 0.9797 for all graphene platforms. The influence of different materials on peak width at half height (W_1/2_) for 12.0 μM catechin was also investigated to correlate the parameter to the selectivity of the materials in the presence of interferences. With the data collated, BDD has the highest W_1/2_ while the other materials have similar W_1/2_ with slight improvement for TRG and NDG as compared to GC.

As observed form the material characterization, TRG showed the lowest content of oxygen functionalities, the highest amount of structural disorders and the largest electroactive surface area. All these factors contributed to improve the material electroanalytical performance, thus resulting in enhanced sensitivity of the electrochemical signal. As for a comparison between the doped graphenes, BDG showed better calibration sensitivity as compared to NDG despite the lowest amount of defects and electroactive surface area presented by the former. Clearly, among the doped graphenes, the electrochemical response is mostly influenced by the kind of heteroatom rather than the properties of the materials. In fact, as recently demonstrated, the favourable thermodynamic interactions between the electron withdrawing boron and the electron donating oxygen groups of the analysed probe strongly influences the oxidation process^15^.

Given the best electrochemical performance of TRG in terms of sensitivity, selectivity and linearity of response, the material was chosen for the application to real sample analysis.

The results obtained for three commercial beer samples and represented as catechin equivalents are depicted in Table 4. The results reveal the dissimilar polyphenol content of the three beer samples due to their composition (relative ratio of malted barley and hops) and brewing process^29^. In addition, a good linearity (R^2^ ≥ 0.9589) and repeatability of results (RSD ≤ 10.44%) were achieved.

Finally, in order to confirm the selectivity of the response towards catechin in real samples, a study on TRG was performed by measuring the concomitant current response of luteolin, another polyphenol present in beer. As it can be seen in Figure S4 (Supporting Information), a significant signal separation of about 120 mV was recorded between catechin and luteolin.

## Conclusions

We investigated the influence of structural properties of doped and undoped graphene materials on their electrochemical performance for the assessment of catechin, a standard polyphenol commonly used as index of food quality. We observed that in general, graphene materials show an enhanced electroanalytical response when compared to bare glassy carbon and boron doped diamond electrodes. This is because of the larger electroactive surface area they possess, together with the sp^2^ network which favours the interactions with the analyte by π-π stacking. As a result of that, an increased intensity of the peak current and a lower oxidation potential was observed on both undoped and doped graphene platforms.

Overall, the undoped graphene namely thermally reduced graphene (TRG) provided the best analytical performance in terms of sensitivity, selectivity and linearity of response due to the intrinsic properties of the material such as lower content of oxygen functionalities, higher amount of structural disorders and larger electroactive surface area as compared to doped graphenes. We demonstrated that in the reported case, the outstanding material properties play a major role towards the oxidation of catechin rather than the nature of heteroatom used for the doping. In addition we found out that within the heteroatom doped materials, the best performance was provided by the boron doped graphene because of the favourable effect of boron in promoting the thermodynamic interactions between the analytical probe and the graphene platform. Finally, we demonstrated the suitability of TRG platform for the real sample analysis by determining the amount of polyphenols, expressed as catechin equivalents, in three commercial beer samples. These findings provide an insight into doped and undoped graphene suitability for food science application.

## Additional Information

**How to cite this article**: Chng, C.E. *et al*. Doped and undoped graphene platforms: the influence of structural properties on the detection of polyphenols. *Sci. Rep.* **6**, 20673; doi: 10.1038/srep20673 (2016).

## Supplementary Material

Supplementary Information

## Figures and Tables

**Table 1:** Characterization of graphene materials by XPS, Raman spectroscopy, and prompt gamma-activation analysis^26^.

Material	C/O ratio	D/G ratio	Amount of dopant
TRG	18.8	1.10	–
BDG	13.5	0.68	23 ppm
NDG	12.9	1.00	6.4 at.% N

**Table 2:** Results for DPV determination of 12.0 μM of catechin on different electrode materials: glassy carbon (GC), boron doped diamond (BDD), thermally reduced graphene (TRG), boron doped graphene (BDG) and nitrogen doped graphene (NDG) electrodes.

Material	Peak Height/μM	Potential/V	RSD%
GC	799.8	0.140	10.1
BDD	11.6	0.563	7.8
TRG	1594.5	0.139	18.0
BDG	1074.5	0.140	19.4
NDG	922.7	0.144	11.4

**Table 3:** 

Material	Slope of Calibration Curve/nA μM^−1^	R^2^	W_1/2_/V
GC	68.267 ± 7.39	0.9448	0.059
BDD	1.695 ± 0.28	0.9957	0.236
TRG	143.220 ± 44.50	0.9797	0.061
BDG	88.283 ± 7.39	0.9895	0.058
NDG	81.282 ± 9.81	0.9905	0.061

Slope of calibration curve, correlation coefficient (R^2^) and peak widths at half height for DPV measurements of catechin on different electrode surfaces: glassy carbon (GC), boron doped diamond (BDD), thermally reduced graphene (TRG), boron doped graphene (BDG) and nitrogen doped graphene (NDG) electrodes. Concentration range: 1.2 – 12.0 μM.

**Table 4:** Catechin equivalents (CE) in beer samples measured by using TRG platform.

Beer samples	CE	RSD (%)	R^2^
Sample n.1	0.972	4.38	0.9964
Sample n.2	1.530	6.01	0.9589
Sample n.3	0.618	10.44	0.9841

CE value was extrapolated by using standard addition method for each beer sample. (CE = milligrams of catechin per 100 ml).
