# Supplementary material for: Doped and undoped graphene platforms: the influence of structural properties on the detection of polyphenols
Source: Sci Rep. 2016 Feb 10;6:20673. doi: 10.1038/srep20673 (PMC4748243; doi:10.1038/srep20673)
Supplement: Supplementary Information [file srep20673-s1.doc]

***Supporting Information***

**Doped and undoped graphene platforms: the influence of structural properties on the detection of polyphenols**

Chu’Er Chng,^a^ Zdenek Sofer,^b^ Martin Pumera^a^ and Alessandra Bonanni^a^*

^a^Division of Chemistry & Biological Chemistry, School of Physical and Mathematical Sciences, Nanyang Technological University, Singapore 637371

^b^Department of Inorganic Chemistry, Institute of Chemical Technology, 166 28 Prague 6, Czech Republic

Fax: (65) 6791-1961
Email: [a.bonanni@ntu.edu.sg](mailto:a.bonanni@ntu.edu.sg)

*Author for correspondence

**Figure S1**. Wide-scan XPS spectra of undoped and doped graphene materials: thermally reduced graphene (TRG); boron-doped graphene (BDG); and nitrogen-doped graphene (NDG).

**Figure S2**. Raman spectra of undoped and doped graphene materials: thermally reduced graphene (TRG); boron-doped graphene (BDG); and nitrogen-doped graphene (NDG). Normalization of spectra with respect to G band was performed for a better comparison.

**Figure S3**. SEM micrographs of graphene materials. A: Nitrogen doped graphene (NDG); B: thermally reduced graphene, undoped (TRG); C: Boron doped graphene (BDG). Scale bar: 1 μm.

**Figure S4**. Differential pulse voltammeric profiles showing the separation of catechin hydrate and luteolin on TRG. Concentration of catechin hydrate added: 0.66 µM. Concentration of luteolin added: 12 µM. Conditions: 100 mM phosphate buffer, pH 7.3; step potential 4 mV, modulation amplitude 25 mV.
